# Strategy Based on Michael Addition Reaction for the Development of Bioinspired Multilayered and Multiphasic 3D Constructs

**DOI:** 10.3390/polym15071635

**Published:** 2023-03-24

**Authors:** Mihaela Olaru, Natalia Simionescu, Florica Doroftei, Geta David

**Affiliations:** 1 “Petru Poni” Institute of Macromolecular Chemistry of Romanian Academy, 41A Gr. Ghica Voda Alley, 700487 Iasi, Romania; 2 Department of Natural and Synthetic Polymers, Faculty of Chemical Engineering and Environmental Protection “Cristofor Simionescu”, “Gh. Asachi” Technical University of Iasi, 71A Bd. D. Mangeron, 700050 Iasi, Romania

**Keywords:** multilayered construct, aza-Michael addition, cryogelation, biocomposite, tissue engineering

## Abstract

The high incidence of osteochondral defects has increased the interest in the development of improved repairing alternatives, with tissue engineering being considered a promising approach. The hierarchical, complex structure of osteochondral tissue requires the design of a biomimetic multilayered scaffold. Here, a multilayered and multiphasic 3D macroporous structure was achieved at subzero temperature by the Michael addition reaction of amino functionalities of collagen with acryloyl groups of a bifunctionalized poly(ε-caprolactone). This green approach has been successfully applied to crosslink layers of different composition, both for their efficient sequential formation and connection. Polyethylenimine functionalized nano-hydroxyapatite (nHAp_LPEI_) was added to the bottom layer. The resulting hybrid cryogels were characterized by morphology, equilibrium swelling ratios, compressive strength analysis, and MTS assay. They presented good stability, integrity, and biocompatibility. The results revealed that the properties of the prepared constructs may be tuned by varying the composition, number, and thickness of the layers. The Young modulus values were between 3.5 ± 0.02 and 10.5 ± 0.6 kPa for the component layers, while for the multilayered structures they were more than 7.3 ± 0.2 kPa. The equilibrium swelling ratio varied between 4.6 and 14.2, with a value of ~10.5 for the trilayered structure, correlated with the mean pore sizes (74–230 µm).

## 1. Introduction

Cartilage and osteochondral defects have a high incidence, and their management remains challenging, mainly due to the complexity of tissues (in terms of composition, structure, and functions) and a wide range of lesions (different types, extent, depth, and location of the injury), which require continuous improvement of their repair strategies. Over time, several clinical treatment methods have been developed, including palliatives (arthroscopic lavage or washout and debridement), reparative (abrasion arthroplasty, drilling and microfracture, and autograft or allograft transplantation), and regenerative treatments (cellular repair by matrix-assisted autologous chondrocyte implantation—MACI).

Nowadays, the tissue engineering approach seems to provide the most promising results, with the other alternatives often presenting risks and serious shortcomings related to side effects [1,2,3]. There are increasing efforts to develop engineered artificial substitutes that best mimic cartilage or osteochondral tissue in terms of composition, architectures, and functions. Research and clinical data have shown that an efficient, integrative cartilage repair requires a stable transition to subchondral bone [3,4,5,6]. To ensure continuity between the synthetic and natural environment, an appropriate substitute must structurally, chemically, and mechanically mimic the mature articular cartilage. With regard to the layered, hierarchical architecture of cartilage and taking into account the importance of the interface between the two distinct tissues, i.e., cartilage and bone, multiphasic (stratified scaffolds with varied composition and mechanical strengths in different layers) and gradient scaffolds were developed. Recently, some of them were commercialized as synthetic acellular cylindrical scaffolds (TruFit™—Smith and Nephew, Smith & Nephew, Andover, MA, USA, MaioRegen™—Finceramica, Faenza, Italy, ChondroMimetic^®^—Collagen Solutions, Glasgow, Scotland, UK, and Agili-C™—CartiHeal Ltd., Kfar Sava, Israel, etc.) [3,4,5,6]. Such complex composite constructs were found to ensure a better integration at the defect site as compared to the tissue-engineered cartilage and, in addition, to allow efficient regeneration of the cartilage, bone, and cartilage–bone interface, being suitable for the repair of osteochondral defects [7,8,9].

The design of the construct from the perspective of composition and architecture is related to the materials and preparative techniques used. These parameters are a key issue in tissue engineering to achieve the main requirements for an optimal scaffold such as biocompatibility, tunable biodegradability, and porous structure (preferably with interconnected pores, suitable for cell attachment and their efficient proliferation) [10]. Practically, all kinds of materials (extracellular matrix (ECM)-based materials, natural and synthetic polymers and their derivatives, ceramic materials, metals, and their combinations in composites) and different fabrication techniques (traditional methods for the preparation of hydrogels and sponges, 3D printing, microfluidic methods, manipulation of the magnetic field in the manufacturing process, combination of scaffold-free and scaffold-based approaches, etc.) have been used for this purpose. Natural polymers (in particular, ECM components, i.e., protein-based and polysaccharides with an important role in the structural and functional maintenance of osteochondral tissue) and composites (mainly containing biodegradable polymers and bioactive ceramics) are applied most often. Natural polymers are usually preferred due to their biocompatibility, biodegradability and, sometimes, to their bioactivity. However, they have serious disadvantages such as weak mechanical properties, sensitivity to temperature and chemicals, uncontrollable degradation kinetics, poor batch reproducibility, and difficulties in purification/sterilization. On the other hand, synthetic polymers provide better control of their mechanical and thermal behavior, degradation rate (regardless of their structure and molecular weight), and processing facilities. Thus, in order to achieve the specific requirements for mimicking the osteochondral tissue, natural polymers are usually modified by crosslinking and/or by combination with synthetic polymers and even bioceramics [1,3,9,11,12,13].

From a structural point of view, hydrogels received much attention for the design of appropriate scaffolds for cartilage tissue engineering [14]. Scaffold porosity is one of the most important design variables for efficient chondrocyte proliferation [13,15]. In the last decade, the potential of cryogel scaffolds for tissue repair has proven that cryotropic gelation [16,17] is one of the main recommended techniques for obtaining appropriate substitutes—highly porous systems with interconnected pores and good mechanical stability. The results demonstrated that constructs of this type (monophasic or biphasic, comprising natural or synthetic polymers) are also excellent candidates for cartilage tissue regeneration [18,19,20,21].

Considering our previous work on the development of cryogel scaffolds [22,23,24], in the present paper we aimed to investigate the efficiency of aza-Michael addition reactions [25] to obtain a multilayered construct, using this green method at subzero temperature both to crosslink collagen-based compositions and to join different layers with variable formulations. The preparation of stable multiphasic scaffolds is challenging and still under considerable investigation. In order to integrate the different layers into a construct, suturing, gluing, simple press fitting, interdiffusion steps, immersion in a suitable crosslinker solution, or more complex strategies are usually applied [4,13]. The use of the Michael addition reaction, i.e., a simple click reaction carried out in mild conditions, with/without a catalyst and without the release of side products, could allow one to avoid common chemical crosslinkers with relative toxicity. For the development of scaffold constructs at high, common, or subzero temperatures, low-molecular crosslinking agents—i.e., diepoxides, glutaraldehyde, water-soluble carbodiimides in the presence of N-hydroxysulfosuccinimide [18,19,20,21,24], or oligomeric bifunctional crosslinkers—have been mentioned in the literature [20,22,23]. The application of the aza-Michael reaction has been reported to prepare degradable, stimuli-responsive cryogels using polyethylene glycol (PEG)-based building blocks and a hyperbranched polyamidoamine dendrimer (PAMAM) with appropriate functionalities [26,27]. To the best of our knowledge, obtaining step-growth collagen-based cryogels via the aza-Michael addition reaction has not been previously reported in the literature. Collagen I (Col I), hyaluronic acid (Hyal), a poly (ε-polycaprolactone) (PCL) derivative (poly (ε-caprolactone) diacrylate—PCL-diA), and surface functionalized nano-hydroxyapatite (nHAp) [24] were chosen for the multiphasic tissue substitute construct. Collagen and hyaluronic acid are the main components of native cartilage [1,2,3], while PCL (a biocompatible, low-cost bioabsorbable synthetic polymer with a low rate of degradation) [28,29] and functionalized nano-hydroxyapatite (nHAp) were chosen in order to improve the mechanical performance and to control the degradation rate of the multiphasic construct [30]. Furthermore, PCL and nHAp are often applied in cartilage and bone tissue engineering, especially as composites, to better mimic the compressive mechanical properties of reparative tissues [3,28,29,31]. The surface functionalization of nHAp also allows a better integration of the inorganic component into the polymer matrix [24]. A number of multiphasic scaffolds comprising collagen I and hydroxyapatite (HAp) in different ratios, i.e., biphasic ChondroMimetic and triphasic MaioRegen systems, have obtained approval for sale [3,5]. Previous preclinical studies [32] have shown that cell-free and cell-seeded scaffolds have similar results in repairing the osteochondral defects. Good clinical results have led to the commercialization of such types of multilayered osteochondral substitutes in the form of acellular scaffolds (sponge form) [33].

In the present paper, the developed cell-free constructs were subjected to the structural characterization and evaluation of the mechanical properties. In addition, the potential use of the obtained cryogels as scaffolds in tissue engineering applications has been verified by cell viability analysis.

## 2. Materials and Methods

Collagen I (Col I, 0.7%, pH—4.5) supplied by Sanimed International Impex S.R.L. (Romania) was concentrated by adding lyophilizate to the appropriate concentration required in synthesis. Dimethylsilanediol hyaluronate (DMSHA, aqueous solution with 0.3% hyaluronan of 1.8–2.2 MDa and 0.3% dimethylsilanediol) was obtained from EXSIMOL S.A.M. (Monaco). Nano-hydroxyapatite surface coated with linear polyethylenimine (nHAp_LPEI_, nanoparticles of 45.5 nm in length and an aspect ratio of 3.91, with a content of 4.5 wt.% LPEI relative to the inorganic material) and poly(ε-polycaprolactone) di-acrylate (PCL-diA, ~ 2.1 kDa) were synthesized before use, according to the literature [24,34]. PCL-diA, obtained as an organic layer, was further purified by precipitation of its solution in dried methylene chloride (Merck) in cold hexane (Merck), dried under vacuum, and stored in a desiccator, in the dark, at 6 °C. Phosphate-buffered saline tablets (PBS, pH 7.4, Sigma Aldrich) and Milli Q ultrapure water were used. All other solvents (acetone p.a. and anhydrous dimethyl sulfoxide—DMSO) purchased from Sigma Aldrich were used as received.

### 2.1. Preparation of Cryogels

The 3D constructs of various compositions were prepared by the cryogelation technique. The multilayered structure was obtained by an iterative strategy. Initially, the collagen solution was concentrated to about 3 wt.% and brought to a pH of 6. According to the envisaged formulation, appropriate amounts of DMSHA solution and nHAP_LPEI_ dispersion (5.2 wt.%) were added under continuous stirring and the pH was adjusted to 7.4 with NaOH (0.1 m) and PBS. A PCL-diA solution (DMSO/acetone 3/2 *v*/*v* mixture) was added at the end. The component volumes for each recipe were calculated to give a final concentration of about 2.4 wt.%. The resulting slurry was further homogenized by ultrasonication for 5 min, deaerated (5 min under vacuum), and cast into cylindrical molds (16 mm in diameter and 10 mm high). After setting the reaction conditions, the monolayered samples comprising only polymers (Col I and PCL-diA) were incubated overnight at −20 °C, followed by 16 h at −12 °C. Then, they were thawed for 2 h at 4 °C, 1 h at 8 °C, and finally at room temperature. After repeated washing with ultrapure water, they were frozen and subjected to lyophilization. For the multilayered structures, the adopted sequential layering strategy imposed the sequential deposition of carefully measured compositions, with the formulations changing in a gradient manner one after the other in the mold, starting with the base layer with the lowest collagen content, followed by pipetting the layers with an increasing percentage of collagen and exposing each one to cryogelation conditions, and finally subjected to thawing, washing, and lyophilization processes. Briefly, for a three-layered construct, a first composition with the lowest collagen content (i.e., 63% relative to total solid content) and the highest amount of nHAp_LPEI_ (20% relative to polymers) was cast into the mold, incubated overnight at −20 °C, followed by 6 h at −15 °C. Then, the top of the first deposited layer was allowed to slightly thaw (to allow partial infiltration of the slurry with the new formulation into the pores of the components of the previous layer, preventing fluid from flowing around the deposited, frozen material), and the intermediary layer dispersion was added with a syringe and frozen according to the same scheme. The assembly was then repeated in the same manner for the addition of the top layer containing collagen, a hyaluronic acid derivative, and PCL-diA. It is recommended that the deposition of the top layer, without mineral content, be carried out at −12 °C. After thawing and repeated washing, the products were frozen and lyophilized. For comparison, a single-layer construct was prepared for each composition under similar conditions. Data for the composition of the layers and constructs are given in Table 1. The different compositions are designated as CHxPyHApz, where C, H, P, and HAp represent collagen, hyaluronic acid derivative, PCL-diA, and nHAP_LPE_; x—weight percent relative to collagen, y—weight percent relative to biopolymers, and z—weight percent of the total amount of polymers in the formulation. To better compare with the cartilage composition [3,6], the collagen percent in the final dry construct was also included in Table 1.

### 2.2. Characterization

The composition and structural modification of the developed constructs for different applied recipes were analyzed by Fourier-transform infrared spectroscopy (FT-IR) in the ATR mode, with a Vertex70 spectrometer (Bruker, Billerica, MA, USA). A scanning electron microscope (SEM instrument—Quanta 200, operating in low vacuum mode) was used to visualize the morphology of the resulting single- or multilayered cryogels, vertically and/or in cross sections of the sheets. The samples were investigated without sputter coating by conducting matter. The average pore size was estimated by taking into account about 100 pores from the registered microphotographs for every investigated sample. The element mapping for the multilayered construct was performed by means of EDX analysis at the surface of the different layers. For the resulting porous products, the swelling ratios (SR) and equilibrium swelling ratios (ESR) were determined using the gravimetric method, in ultrapure water, at 25 °C. The calculation was made by considering the difference between the weight of the hydrogels after and before swelling divided by the initial weight of the sample in the dried state. The results were expressed as the average of three different measurements for each type of examined sample.

Unconfined compression tests were performed on cylindrical samples preliminarily swollen in PBS for 4 h (15 mm in diameter and approx. 6.5 mm in height) with a Brookfield Texture PRO CT^®^ Analyzer at room temperature, with a load cell of 4500 g and a speed test of 0.2 mm/s up to 50% deformation. The elastic modulus (*E*) was calculated from the tangent slope of the stress-strain curve in the range from 15% to 30% deformation, according to the equation

E=σε=FAΔll0

where *σ* is the compressive stress, *ε* the strain, *F* the force (N), *A* the cross-sectional area of the hydrogel (m^2^), Δ*l* the length change, and *l*_0_ the original length.

Three different specimens were evaluated for each 3D construct type, and the final values were expressed as the average of the measurement values ± standard deviations.

### 2.3. In Vitro Biocompatibility Assessment (MTS Assay)

Human gingival fibroblasts (HGF, CLS Cell Lines Service GmbH, Eppelheim, Germany) were seeded into 96-well tissue, culture-treated plates at a density of 0.5 × 10^5^ cells/mL and allowed to adhere overnight in complete cell culture medium: MEM α medium with 10% fetal bovine serum (FBS, both from Gibco, Thermo Fisher Scientific, Waltham, MA, USA) and 1% Penicillin-Streptomycin-Amphotericin B mixture (10 K/10 K/25 μg, Lonza, Basel, Switzerland). Extracts were done for every investigated sample in complete cell culture medium at 10 mg/mL, for 24 h, at 37 °C. The CellTiter 96^®^ AQueous One Solution Cell Proliferation Assay (MTS, Promega, Madison, WI, USA) was used to assess the biocompatibility of samples’ extracts, according to the manufacturer instructions and ISO 10993-5:2009(E). Cells were incubated for 24 h with fresh, complete medium (Control) or different concentrations of samples’ extracts (2.5 mg/mL, 5 mg/mL, 7.5 mg/mL, and 10 mg/mL). After incubation with MTS reagent (3 h), absorbance readings were done at 490 nm on a FLUOstar^®^ Omega microplate reader (BMG LABTECH, Ortenberg, Germany). Experiments were done in triplicate, and treated cell viability was expressed as a percentage of the Control cells’ viability (means ± standard deviation). Data were statistically analyzed by the independent two-tailed (Student’s) *t*-test, considering *p* < 0.05 to be statistically significant.

## 3. Results

### 3.1. Influence of Polymerization Conditions on Collagen–PCL Construct Properties

**Scheme 1:**
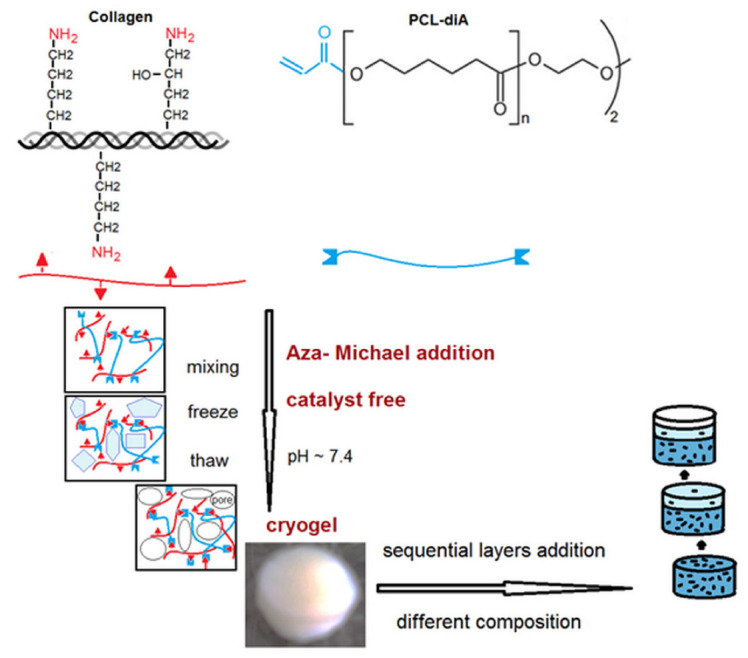
Schematic representation of the cryogels’ synthesis strategy.

A series of single and multilayer constructs were obtained by applying the aza-Michael addition reaction between collagen and PCL-diA and the cryogelation process (Scheme 1).

**Figure 1:**
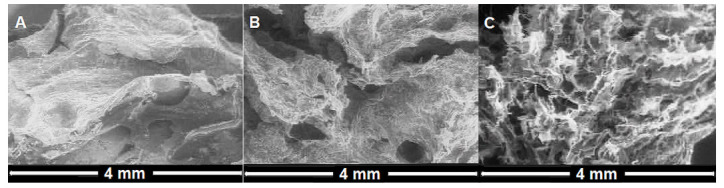
Characteristic micrographs (typical microstructures) for the CP7 sample prepared under different conditions: (**A**) 37 °C, final dispersion concentration 1.25%, (**B**) −12 °C, final dispersion concentration 1.25%, and (**C**) −12 °C, concentration of reaction medium 2.4%.

In order to establish the appropriate polymerization conditions and correlate them with the construct characteristics, a first experiment was developed using a similar recipe (comprising collagen in an aqueous solution of pH~7.4 and PCL-diA) for the reaction under the usual conditions (37 °C) and at sub-zero temperature. Considering our previous work [22], a temperature of −12 °C and a concentration of 1.4 wt.% were fixed for cryogelation. Taking into account that biomedical applications were envisaged, no catalyst was added. The reaction time for cryogel fabrication was extended to 16 h to allow for efficient crosslinking, a lower temperature implying a decrease in the reaction rate. The resulting products did not show proper morphology and mechanical properties. The sample synthesized at 37 °C was obtained as a friable monolith, while the cryogel resulted in a highly elastic, unstable material, presenting a random microstructure with mainly large (approx. 400 µm) and isolated pores, and thick walls. By increasing the concentration of the initial dispersion to 2.4 wt.%, a stable, elastic, and more homogeneous construct with interconnected and evenly distributed pores in the range from 100 to 340 µm (sample 2, 230 μm average value, Table 1) was obtained (Figure 1). The effect of the concentration of the reaction mixture and of the applied preparative protocol is evident.

**Figure 2:**
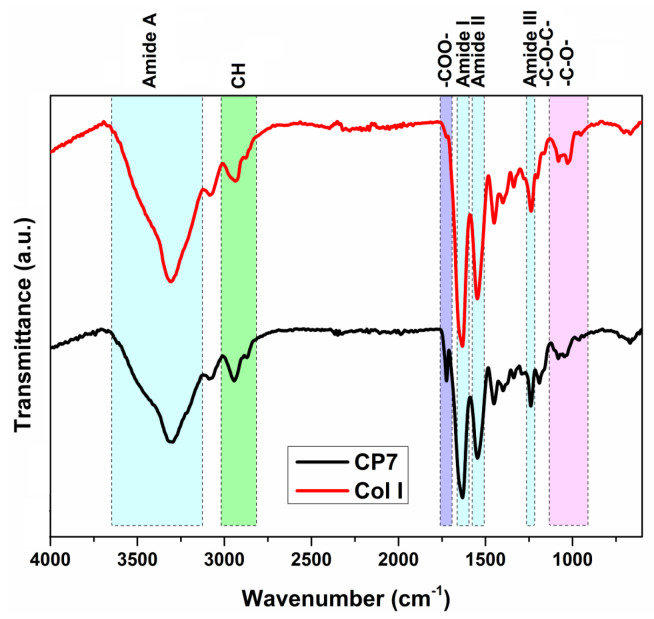
FTIR spectra of commercial collagen and of the CP7 sample (Entry 2, Table 1).

Figure 2 illustrates the FT-IR spectra of commercial type I collagen and of sample 2 (cryogel CP7, Table 1). The FTIR spectrum of type I collagen (Col I, Figure 2) shows a series of characteristic absorption bands. Thus, the bands from 3309 cm^−1^ (amide A) and 3081 cm^−1^ (amide B) can be associated with the stretching vibrations of N–H groups involved in intermolecular hydrogen bonds, and the two absorption bands from 2936 cm^−1^ and 2875 cm^−1^ can be attributed to asymmetric and symmetric CH stretching vibrations from the collagen structure [35]. The main band related to the amide III structure (ascribed to a combination of the C–N stretching vibration, the in-plane deformation of N–H groups, and the wagging vibrations of CH_2_ units from glycine backbone and proline side chains [36]) can be observed at 1236 cm^−1^. Other absorption bands can be observed at 1633 cm^−1^ (amide I band, attributed to stretching vibrations of the peptide C = O group), 1545 cm^−1^ (amide II band—a combination of C–N stretching and N–H bending in triple helix), 1451 cm^−1^ (wagging vibration of CH_2_ groups from pyrrolidine units found in proline and hydroxyproline residues), 1338 cm^−1^ (vibration of CH_2_ side groups), and 1024–1079 cm^−1^ (large or small band due to the stretching vibration of C–O group in carbohydrate residues). The FT-IR spectrum of the CP7 cryogel/sample 2 (Figure 2) shows modifications, i.e., a decrease in the amide A band due to the involvement of amine groups in the reaction with PCL-diA, an increase and enlarging of the band at 2944 cm^−1^ attributed to the contribution of CH_2_ groups from PCL, and the appearance of the band situated at 1724 cm^−1^, related to the stretching vibration of the C = O group in the polyester. The evaluation of the integrity of the collagen triple helix, a parameter that can be correlated with the maintenance of bioactivity and the lack of its denaturation, was performed by analyzing the absorption ratio of the amide III band vs. the band from 1451 cm^−1^ [37]. A value of this ratio of 1 is related to a fully triple helical conformation. The main component of the amide III band situated at around 1240 cm^−1^ is attributed to the specific-N inter-strand hydrogen bonds from the triple helix [36]. In the present study, the A_III_/A_1451_ cm^−1^ ratio is 0.89 for Col I and 1.03 for sample 2 (CP7), which implies the preservation of the native collagen structure.

**Figure 3:**
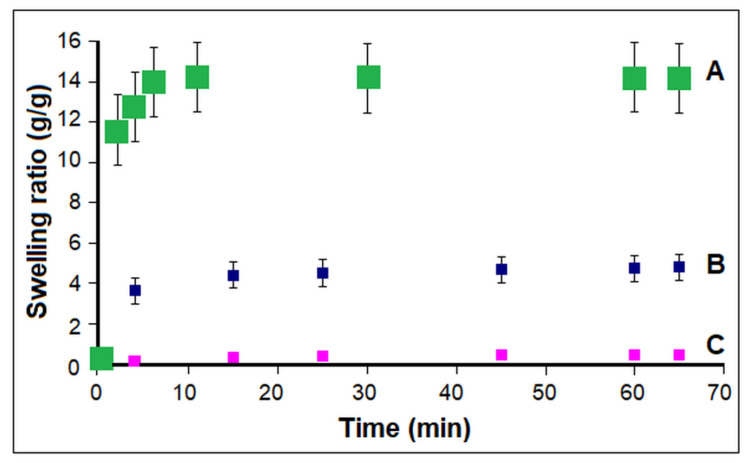
Swelling behavior: effect of the synthesis conditions for the CP7 sample (Entry 2, Table 1) obtained at different temperatures and feed concentrations: (A) −12 °C, c—2.4%; (B) −12 °C, c—1.25%; (C) 37 °C, c—1.25%.

The macroporous structure with interconnected pores of cryogel 2 (CP7, Table 1) led to a significant and rapid swelling process as compared to the samples prepared at a lower concentration of components in the reaction environment (Figure 3). A rapid increase in water absorption to equilibrium (approx. 1–3 min) can be observed in the initial stage for cryogel 2 obtained for a concentration of 2.4 wt.% of the reactants, while sample 1 synthesized at a concentration of 1.25 wt.% reached a steady state after about 7 min. The corresponding conventional hydrogel prepared at room temperature and low concentration, obtained as a rigid material, reached the equilibrium swelling after a longer time (15 min) and showed a much smaller equilibrium swelling ratio (0.47 ± 0.02) than homogeneous cryogel 2 (ESR−14.0 ± 0.3) and even than cryogel 1, obtained for a similar concentration in feed (ESR-4.8 ± 0.08). In addition, due to a high tendency of fragmentation, the investigated rigid sample turned into small pieces when removed from the aqueous solution for weighing, after at least 75 min.

**Figure 4:**
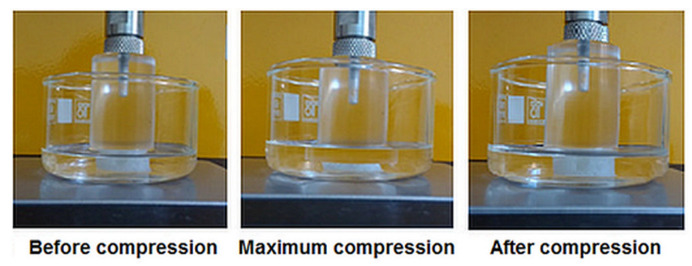
Real-time imaging of sample cryogel CP7 (Entry 2, Table 1) during the compression test.

**Figure 5:**
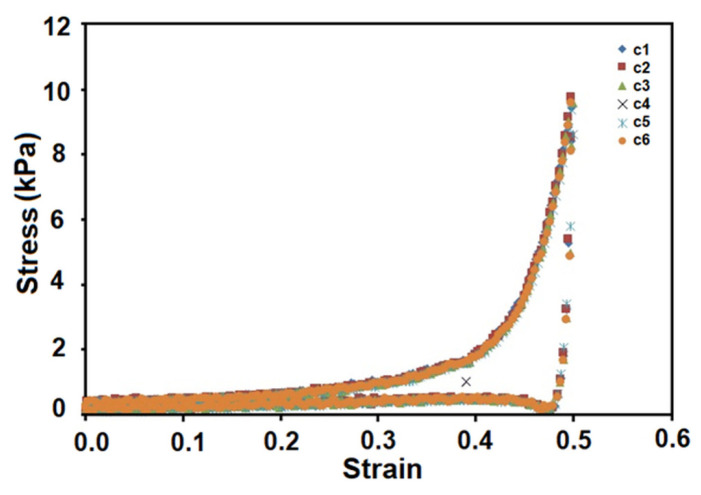
Cyclic compressive properties of sample CP7 (Entry 2, Table 1).

The compression test revealed high stability and elasticity (3.5 ± 0.02 kPa) for sample 2, specific to cryogel structures. The real-time imaging of sample cryogel CP7 before, during, and after compression tests can be seen in Figure 4. Two distinct stages of deformation can be observed in the stress-strain curves of the tested cryogel 2 (CP7) (Figure 5). Up to 30% strain, the cryogel exhibits linear elasticity (attributed to pore wall bending), indicating that, within this low strain range, the macroporous structure remains stable in terms of mechanical properties. The increase in slope in the 30–60% strain range can be correlated with the deformation of the macroporous structure due to pore densification under pressure. The relatively smooth increase in slope in the 30–50% strain range may suggest that this strain process occurs at a relatively low level as compared to the process occurring in the 50–60% strain range. After unloading, the sample quickly recovers its initial shape and size even after 6 compression cycles and shows no signs of cracking. The compression stress-strain graph of cycle 6 is identical to that of cycle 1 (Figure 4 and Figure 5).

### 3.2. Multilayered Structures Obtained by Aza-Michael Addition

**Figure 6:**
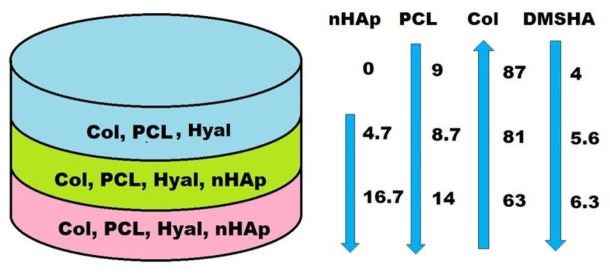
Schematic representation of the typical compositional (wt.%) variation in a trilayered construct (sample TL1).

To increase the design biomimicry, we further attempted to obtain multilayer constructs by using aza-Michael addition for both intra-layer crosslinking of the components and the inter-layer connection. Bilayered (BL-containing layers with formulations 3 and 4) and trilayered constructs (TL1-comprising layers with formulations 5, 6, and 7, CH10P20HAp20/ CH7P10HAp5/CH5P10 at a volume ratio of 9/5/6; and TL2-comprising layers with formulations 5, 8, and 9, CH10P20HAp20/CH10P15HAp5/CH7P10 at a volume ratio of 9/6/4) were prepared. For the trilayered structures, the amount of collagen decreased from the superficial area towards the bottom, corresponding to the calcified zone (i.e., from 86.6% to 63.1% for TL1, Table 1), according to the natural tissue composition [3,6], while the content of proteoglycan, PCL, and nHAp content showed an opposite trend, i.e., increasing from 4%, 9%, and 0% to 6.3%, 14%, and 16.7%, respectively (Figure 6). The weight percent of nHAp_LPEI_, as well as the amount of PCL-diA, was increased in the bottom layer in order to provide a higher resistance to compression forces. The resulting material presents suitable mechanical characteristics because of the good inherent properties of both nHAp_LPEI_ and PCL-diA components, as well as the interactions of PCL-diA with LPEI coverage of nHAp. The presence of LPEI requires a higher amount of polyester diacrylate, even if the collagen weight percent (main reactant in the aza-Michael addition) is the lowest in the bottom layer of the designed construct. Thus, the design of the construct is closer to MaioRegen and ChondroMimetic commercial osteochondral substitutes, known for their high clinical efficiency. MaioRegen osteochondral substitutes are marketed as 3D bi- and trilayered structures based on Mg-HAp nanocrystals nucleated on collagen fibers, having an upper layer of type I equine collagen, a second layer comprising 70% type II collagen and 30% Mg-Hap, and, eventually, a third layer comprising 30% collagen and 70% Mg-HAp in order to mimic the subcondral bone. The ChondroMimetic product is a bilayered cylindrical implant with an approx. 2-mm-thick cartilage-like upper layer of collagen and glycosaminoglycan and an approx. 10-mm-thick bone-like lower layer comprising collagen, calcium phosphate, and GAG.

**Figure 7:**
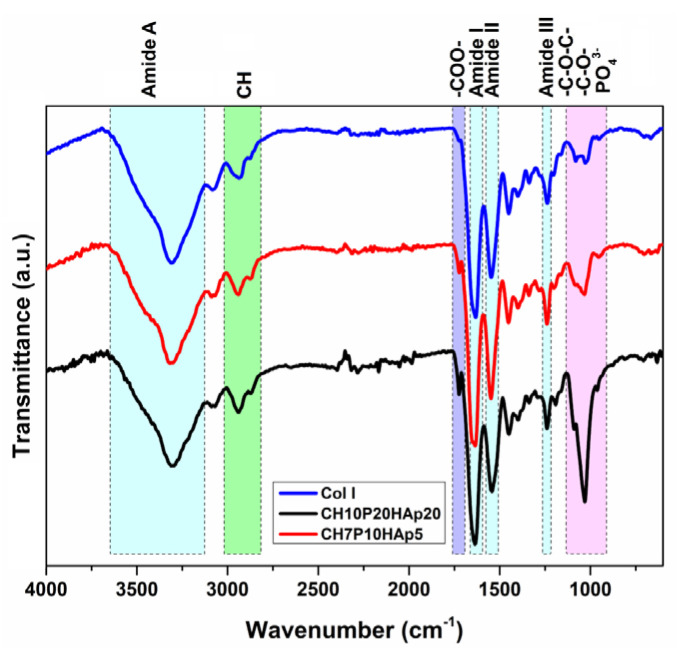
Typical FTIR spectra of the included construct layers of different compositions.

The FTIR spectra of the multilayered structures (Figure 7) are characterized by the presence of the typical vibration peaks of Col I, PCL, hyaluronan, and HAp, demonstrating the structural integration of the recipe components into the construct network. The absorption band corresponding to the amide I band of collagen (1633 cm^−1^) registered a slight shift to lower wavelengths (i.e., a shoulder at 1650 cm^−1^ for the intermediate CH7P10HAp5 layer). Usually, the modification of amide I absorption can be attributed to changes in collagen protein chain conformations, variation in supramolecular organization or an increase in the number of hydrogen bonds, and an increase in the band intensity, typically being correlated with an increase of the structural order [35]. The evaluation of the integrity of the collagen triple helix, carried out by analyzing the absorption ratio AIII/A_1451_, evidenced a variation of its value from 0.89 (initial collagen) to values of 0.98 for the bottom layer (CH10P20HAp20), 1.08 for the intermediate layer (CH7P10HAp5), and 1.09 for the top layer (CH5P10), respectively. The increase of this ratio, which is considered an index for evaluating the collagen structural integrity [38], indicates that the triple-helix structure of the collagen crosslinked with PCl-diA and exposed to the cryogelation process was not changed. In addition, the upward trend indicates the improvement of the order level in such types of composite materials. The difference between the frequencies of amide I (varying from 1631 cm^−1^ to 1636 cm^−1^) and II (varying from 1541 cm^−1^ to 1550 cm^−1^) is < 100 cm^−1^, also indicating the maintenance of the triple-helix structure [39]. In addition, a decrease in the intensity of the band from 1545 cm^−1^ (amide II, N–H stretching, and C–N deformation) was observed, more pronounced in the case of the intermediate layer, as well as the reduction of the amide A band. They can be correlated with the reduction in the number of primary amine groups due to the Michael addition reaction with the acryloyl groups of bifunctionalized poly(ε-caprolactone).

**Figure 8:**
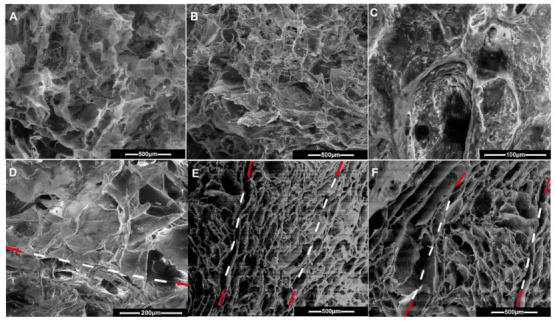
Representative SEM images for the prepared cryogels (mono-, bi-, and trilayered): (**A**) sample 2/CP7, (**B**) and (**D**) bilayered constructs at the interface CH5P15HAp20/CH5P10HAp5, (**C**) sample 3/CH5P15HAp20, (**E**) TL1 trilayered sample (CH10P20HAp20/CH7P10HAp5/CH5P10), and (**F**) TL2 trilayered sample (CH7P10/CH7P10HAp5).

The level of crosslinking was increased by increasing the content of PCL-diA relative to Col I, by adding proteoglycan that was able to act as a crosslinker (due to development of specific electrostatic interactions and H bonds) [40] and polyethylenimine-coated nHAp, thus affecting the morphology, swelling, and mechanical behavior of the 3D structures. During cryogelation, the aza-Michael addition reaction occurred at a temperature below the freezing point of the reaction solution (mixture of water, DMSO, and acetone), thus creating an apparently frozen system consisting of ice crystals and microchannels of unfrozen liquids. The crystals formed as the solvent mixture freezes acted as irregular pore formers, with the generation of an interconnected porous structure consisting of both isolated and large interconnected pores. As much of the solvent mixture freezes, the components used in making these cryogels accumulated in the unfrozen liquid microphase (microchannels) and formed the crosslinked walls around the crystals. As evidenced by SEM image analysis, Figure 8, a decrease in average pore sizes from 230 µm (sample 2/CP7) to 112–131 µm (samples 7 and 9), 118 µm (sample 4), 95 µm (sample 6), 85 µm (sample 8), 76 µm (sample 3), and up to 74 µm (sample 5) was noticed. Thus, layers with different pore sizes can be easily obtained by choosing the appropriate recipe composition. The inclusion of nHAp along and between the collagen fibrils, coated with PCL, can be clearly observed in Figure 8C. This aspect agrees with the increased ordering level observed by the FTIR spectra for samples 5 and 6 (CH10P20HAp20 and CH7P10HAp5, Table 1). By including glycosaminoglycans and nHAp_LPEI_, as well as by increasing the PCL content, the pore walls became thicker from the top to the bottom. The reduced reaction rate due to the low temperature imposed the extension of the reaction time to allow the complete reaction for the fabrication of the cryogel, especially of the multilayered construct. Keeping the construct layers for a longer period of time at sub-zero temperatures allowed the morphology to change (as previously observed [22]), with the appearance of a fibrillar collagen structure in the bottom layer (Figure 8B–D). To allow the successful deposition of the next layer and to avoid a subsequent delamination, the new formulation must partially infiltrate into the pores of the previously formed layer. The upper part of the previous layer must be only partially thawed before the deposition of the next layer to allow the interpenetration without surrounding the deposited material with the new dispersion. According to this synthesis protocol, a good interface connection could be achieved (Figure 8B–F) using the unreacted amino groups from the top of the previous layer. As follows, the use of a syringe for the deposition on the top layer can facilitate the deposition of the top layer parallel to the surface and the generation of large channels. Thus, the pores become cylindrical while remaining interpenetrated. The high porosity and interconnected pores of the multilayered constructs create adequate physical space to facilitate the movement and distribution of cells throughout the structure.

The difference in the composition of the layers included in the TL1 construct was evidenced by mapping the elements using EDX analysis (Table 2).

**Figure 9:**
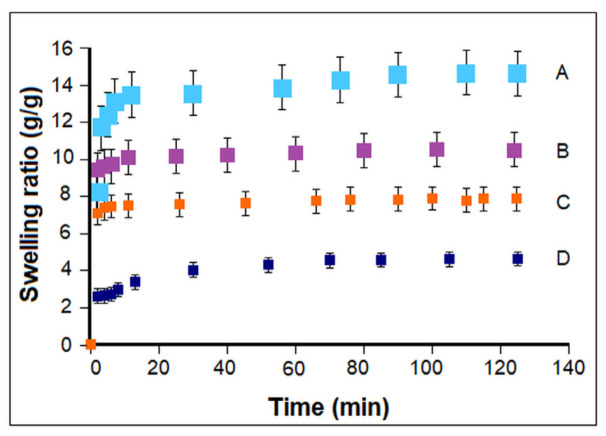
Swelling kinetics of the prepared cryogel constructs for different composition, morphology, and architecture: (**A**) sample 7 (CH5P10), (**B**) TL1 trilayered sample (CH10P20HAp20/CH7P10HAp5/CH5P10), (**C**) bilayered structure comprising formulations 5 and 6 (CH10P20HAp20/CH7P10HAp5), and (**D**) sample 5 (CH10P20HAp20).

As expected, the variation of composition and morphology from the bottom to the top layers gave rise to an increase in the swelling ability (Figure 9) and in the flexibility of the layered constructs (Table 3). The swelling behavior reflects the water absorption capacity of a material, i.e., the penetration of water into the pores (until they are completely filled) through the diffusion phenomenon. The swelling behavior is related to the degree of crosslinking, the network microstructure, hydrophilicity of the material, and morphological characteristics (presence and stability of a porous structure in water and filling of pores by the solvent) [41]. For the bottom layer, the high content of PCL-diA, DMSHA, and nHAP_LPEI_ in the formulation resulted in a higher crosslink density, less free hydrophilic groups due to their involvement in the corresponding interactions, and smaller pores, with dense and thick walls. Finally, a lower water absorption capacity and a lower swelling rate were registered. Considering the data reported here, one can optimize the design of such multilayered constructs mimicking the osteochondral tissue by tailoring the layers’ composition, number, and thickness. To compare, a value of ESR of 7.5 was mentioned in the promotional literature [42] for the multilayered osteochondral substitute commercialized by MaioRegen. This lower value can probably be connected with the higher HAp content.

The mechanical strength of a scaffold is a very important parameter in cartilage tissue engineering, providing integrity to the chondrocytes to grow without deformation and to withstand the mechanical load if the scaffold has to be implanted in the joint to replace the damaged tissue. A functional scaffold must possess mechanical properties correlated with suitable porosity, thus providing a sequential transition in which the regenerated tissue assumes its function as the scaffold degrades. With the increase in the mineral content and PCL-diA in the feed formulation, an enhancement of the mechanical properties such as stiffness and strength was observed. The bottom layer is rigid and friability susceptible, depending on the composition. Elasticity is increased by adding upper layers, with less or no mineral content, and thus cracking is avoided (which is likely to occur in the base layer with high HAp and PCL content after compression). No delamination was observed during swelling or even after 4 compression cycles for the multilayered constructs. The results of the compression test are summarized in Table 3, demonstrating the feasibility of adjusting the mechanical properties of the constructs by layering. TL1 has a slightly higher E value than TL2, probably because TL1 was subjected to a repeated freezing-thawing cycle in the final preparation step.

The obtained values of the elastic modulus are low compared to the range of characteristics of human articular cartilage tissue, but they are similar to the values reported for other scaffolds based on natural polymers [43]. An improvement could be obtained by double crosslinking, i.e., by combining with a low molecular crosslinker.

**Figure 10:**
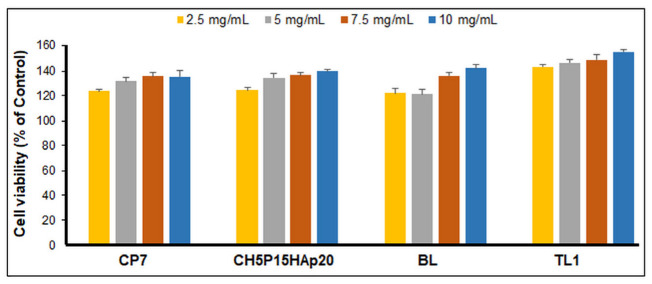
Cell viability of normal fibroblasts exposed to different samples’ extracts at various concentrations in complete cell culture medium (2.5 mg/mL, 5 mg/mL, 7.5 mg/mL, and 10 mg/mL) for 24 h.

In vitro biocompatibility was assessed with MTS assay for samples 2 (CP7), 3 (CH10P15HAp20), BL, and TL1 after 24-h incubation of human gingival fibroblasts (HGFs) in their extract’s solutions (corresponding to 2.5 mg/mL, 5 mg/mL, 7.5 mg/mL, and 10 mg/mL). The results showed that samples’ extracts were not cytotoxic for HGFs at the tested concentrations (Figure 10). A dose-dependent stimulation of the HGF proliferation process was observed for all samples during the 24-h incubation (lowest vs. highest concentration: *p* < 0.01 for CP7, *p* < 0.001 for CH10P15HAp20, *p* < 0.001 for BL, and *p* < 0.001 for TL1), the highest cell viability being obtained for the trilayered construct TL1 (*p* < 0.001 vs. CP7, CH10P15HAp20, and BL, respectively). This behavior can be ascribed to a synergistic effect of the combination (in different amounts) of collagen and a hyaluronic acid derivative, which favored obtaining an optimal environment for cell survival, acting as biological cues. They are the first to support partial degradation in the environmental medium [30].

## 4. Conclusions

The cryo-aza-Michael addition method (green synthesis) has been successfully applied for the preparation of porous stratified composite scaffolds, mimicking the cartilage architecture, using mainly collagen type I, poly-ε-caprolactone diacrylate, a hyaluronic acid derivative, and polyethylenimine-coated nHAp, gradually disposed in different construct layers. The results obtained for the fabrication, the comparative characterization, and the compressive mechanical measurements for the developed mono-, bi-, and trilayered constructs showed that the physic-mechanical properties could be tuned by adjusting the composition, mainly the collagen/PCL-diA ratio (amine and acrylate groups stoichiometry) and the mineral content. Generally, the integrity of the multilayered structures was maintained after successive swelling or compression. It has been proven that the prepared cryogels are highly biocompatible. The triple-helix conformation in collagen is preserved regardless of the cryogel/layer formulation The presented data recommend such 3D biomimetic structures as promising materials for biomedical applications, i.e., (osteochondral) scaffolds or platforms for drug screening.

## Figures and Tables

**Table 1:** Feed composition of the synthesized cryogels.

Entry	Code	DMSHA ^a^	PCL-diA ^b^	HAp_LPEI_ ^c^	Col ^d^	Dispersion Concentration ^e^
		(wt.%)	(wt.%)	(wt.%)	(wt.%)	(wt.%)
1	CP7	-	7	-	93.4	1.25
2	CP7	-	7	-	93.4	2.40
3	CH5P15HAp20	5	15	20	69.0	2.90
4	CH5P10HAp5	5	10	5	82.5	2.30
5	CH10P20HAp20	10	20	20	63.1	2.45
6	CH7P10HAp5	7	10	5	80.9	2.30
7	CH5P10	5	10	-	86.6	2.40
8	CH10P15HAp5	10	15	5	75.3	2.30
9	CH7P10	7	10	-	85.0	2.25

^a^ relative to collagen, ^b^ relative to biopolymers, ^c^ relative to total polymers, ^d^ concentration of collagen in the final construct, and ^e^ total organic and inorganic material included in the aqueous dispersion (protein + glycosaminoglycan derivative + PCL-diA + nHAp_LPEI_).

**Table 2:** EDX data for the trilayered construct TL1.

	Top Layer (Entry 7)		Middle Layer (Entry 6)		Bottom Layer (Entry 5)	
Element	wt. (%)	at. (%)	wt. (%)	at. (%)	wt. (%)	at. (%)
C	40.7	46.1	41.3	46.9	43.7	49.9
N	28.0	27.2	26.2	25.6	21.5	21.1
O	31.3	21.6	32.0	27.3	32.9	28.2
P			0.2	0.1	0.7	0.3
Ca			0.3	0.1	1.2	0.4

**Table 3:** Equilibrium swelling ratios (ESR) and Young’s modulus values of the investigated samples.

Composition/Architecture	ESR	E (kPa)
CP7/1	4.8 ± 0.08	-
CP7/2	14.0 ± 0.3	3.5 ± 0.02
CH5P15HAp20	5.6 ± 0.2	9.6 ± 0.7
BL	7.6 ± 0.1	8.9 ± 0.5
CH10P20HAp20	4.6 ± 0.2	10.0 ± 0.6
TL1	10.5 ± 0.3	8.1 ± 0.1
TL2	9.1 ± 0.3	7.3 ± 0.2

## Data Availability

The data presented in this study are available on request from the corresponding author.
